# Modelling individual variation in human walking gait across populations and walking conditions via gait recognition

**DOI:** 10.1098/rsif.2024.0565

**Published:** 2024-12-11

**Authors:** Kayne A. Duncanson, Fabian Horst, Ehsan Abbasnejad, Gary Hanly, William S. P. Robertson, Dominic Thewlis

**Affiliations:** ^1^ Adelaide Medical School, The University of Adelaide, Adelaide 5005, Australia; ^2^ Department of Training and Movement Science, Johannes Gutenberg-University, Mainz 55122, Germany; ^3^ Australian Institute for Machine Learning, The University of Adelaide, Adelaide 5000, Australia; ^4^ Information Sciences Division, Defence Science and Technology Group, Edinburgh 5111, Australia; ^5^ School of Electrical and Mechanical Engineering, The University of Adelaide, Adelaide 5005, Australia

**Keywords:** gait recognition, gait, walking speed, footwear, sex, cross-dataset

## Abstract

Human walking gait is a personal story written by the body, a tool for understanding biological identity in healthcare and security. Gait analysis methods traditionally diverged between these domains but are now merging their complementary strengths to unlock new possibilities. Using large ground reaction force (GRF) datasets for gait recognition is a way to uncover subtle variations that define individual gait patterns. Previously, this was done by developing and evaluating machine learning models on the same individuals or the same dataset, potentially biasing findings towards population samples or walking conditions. This study introduces a new method for analysing gait variation across individuals, groups and datasets to explore how demographics and walking conditions shape individual gait patterns. Machine learning models were implemented using numerous configurations of four large walking GRF datasets from different countries (740 individuals, 7400 samples) and analysed using explainable artificial intelligence tools. Recognition accuracy ranged from 52 to 100%, with factors like footwear, walking speed and body mass playing interactive roles in defining gait. Models developed with individuals walking in personal footwear at multiple speeds effectively recognized novel individuals across populations and conditions (89–99% accuracy). Integrating force platform hardware and gait recognition software could be invaluable for reading the complex stories of human walking.

## 1. Introduction

Human walking is a multi-faceted behaviour that manifests through the complex interplay of multiple organ systems [1,2]. As such, variations in the walking pattern (i.e. gait) reflect underlying biological variation among individuals, groups, communities, subpopulations and populations [3–7]. Insights into the relationships between gait and biological state can inform multiple application domains, with healthcare and security being the most researched [8,9]. In healthcare, the aim is to use gait as a personal functional marker to assist in the management of neurological and musculoskeletal pathology [10]. In security, the aim is to use gait as a personal biological trait (i.e. biometric) to assist in person recognition in dynamic settings [11]. Despite both domains requiring an understanding of gait variation, studies in each domain predominantly employ distinct methods.

Gait analyses in healthcare mostly prioritize measurement quality and diversity, whereas gait analyses in security mostly prioritize statistical complexity. In healthcare, gait measurements are typically taken using multiple specialized instruments (e.g. three-dimensional motion capture cameras, force platforms and electromyography sensors) to acquire detailed and holistic representations of gait [12]. Systematic reviews show that datasets used in studies oriented towards healthcare are generally small and confined in terms of population sample (e.g. less than 40 individuals from a specific population sample and narrow demographic) and walking conditions (e.g. a single session of barefoot walking at self-selected preferred speed) [13,14]. Gait variation is typically analysed at the group level by performing multiple comparisons across discrete univariate statistical models. For example, it is common to compare specific features (i.e. measurable characteristics) of the gait pattern (e.g. spatio-temporal, kinematic and kinetic parameters) within or between groups defined by a demographic attribute, pathology or treatment strategy [14–17]. To facilitate more personalized care that suits the needs of individual patients, there is a demand to analyse individual-level variation in entire gait patterns using multivariate statistical methods such as artificial intelligence (AI).

In the security domain, gait analysis could be helpful for authentication (e.g. smart homes), surveillance (e.g. airport security) and forensics (e.g. suspect detection). Studies on gait-based person recognition (also known as gait recognition or gait re-identification) typically use commercial off-the-shelf vision sensors such as red green blue (RGB), infrared, or depth cameras because they are convenient and unobtrusive [9]. The review by Singh *et al*. shows that most datasets used for gait recognition are relatively large and diverse but contain low-resolution video footage of two-dimensional body appearance [18]. Gait recognition requires individual-level modelling to detect features of gait that differ between individuals (maximal inter-individual variation) yet remain consistent within individuals over time (minimal intra-individual variation). Hence, most studies focus on developing complex multivariate models such as deep neural networks to disentangle identifying features of gait from features relating to body appearance [9,19,20]. Given that gait analysis methods in healthcare and security appear complementary, it could be beneficial to combine their strengths.

One way to combine the strengths of methods from each domain to better understand gait variation is to develop AI models for gait recognition using force platform data. Force platforms measure the magnitude and point location of force at the ground in response to force applied through the feet (ground reaction force and centre of pressure, respectively). They are convenient and unobtrusive to deploy yet provide gait data that is high quality, information rich, low dimensional (resource efficient), interoperable and privacy preserving (not naturally identifiable to the human eye) [12,13,21]. Prior works have indicated that machine learning gait recognition models developed on force platform data could inform both healthcare and security. Horst *et al*. highlighted the potential of conducting force platform-based gait recognition using explainable artificial intelligence (XAI) methods (tools that assist in explaining the learning, decision-making and performance of complex and opaque AI models) for personalized clinical gait analysis [3,22,23]. Models were developed on data acquired in controlled experimental conditions (e.g. a single session of barefoot walking at self-selected preferred speed) and then interrogated using an XAI method known as layer-wise relevance propagation (LRP). LRP highlighted portions of ground reaction force patterns that were used to recognize individual participants based on the notion that unique force features could reflect current or incipient pathology [22]. Another recent line of work showed the promise of force platform-based gait recognition models for security applications using data acquired in less-controlled experimental conditions [21,24]. In particular, Duncanson *et al*. found models to be highly effective at recognizing individuals walking in personal footwear at multiple speeds but noted a vulnerability to changes in these factors within individuals over time [24]. Collectively, these studies provide preliminary evidence for the broad utility of force platform-based gait recognition models.

**Figure 1.**
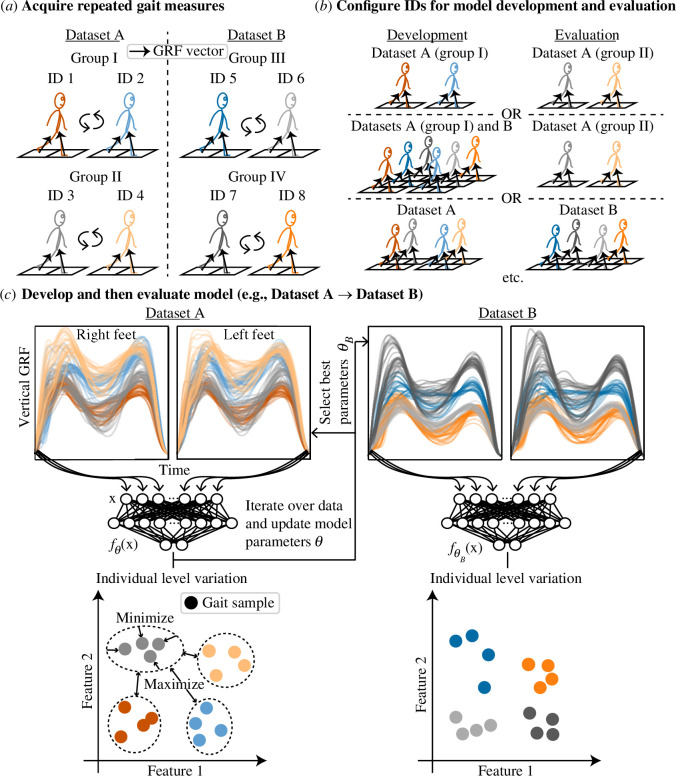
Simplified illustration of gait recognition using multiple force platform gait datasets. (*a*) Force platforms measure variables pertaining to the ground reaction force (GRF). Force platform gait datasets are typically acquired by having participants walk repeatedly over consecutive force platforms embedded in a walkway. (*b*) There are different ways of configuring individuals (IDs) from multiple datasets for model development and evaluation. (*c*) In this example, separate datasets are used for model development compared with model evaluation. A neural network model $f_{\theta }(x)$ learns parameters θ that extract features from the input x (vertical GRF) that distinguish between IDs from dataset A. The version of the model that encodes the most distinguishing features ($f_{\theta_{B}}(x)$) is evaluated on IDs from dataset B to infer the extent to which distinguishing features of gait are common between datasets.

All prior studies using machine learning for force platform-based gait recognition developed (trained and validated) and evaluated (tested) models on either the same individuals (subject-dependent configuration) or different individuals from the same dataset (subject-independent, same-dataset configuration) [9]. This is a significant limitation because appraisals of model performance, and thus the degree of variation in gait, could be biased towards a specific demographic or set of experimental conditions. The first aim of this study was to determine if gait recognition performance differs depending on the dataset(s) used for model development and evaluation using different configurations of four large force platform gait datasets from around the world (AIST [25], Gutenberg [26], GaitRec [27] and ForceID-A [24]). The second aim was to examine if demographic attributes and experimental conditions help to define gait variation using XAI. To fulfil these aims, a novel gait analysis method was proposed that enables simultaneous characterization of individual, group and dataset-level variation in gait patterns (figure 1). The hypotheses were as follows:

1. Adding data from external datasets for model development will improve performance by giving models access to additional information on what makes gait differ between individuals. For example, following the notation ‘development dataset(s) → evaluation dataset’, performance will be better in the ForceID-A and AIST → ForceID-A configuration than the ForceID-A → ForceID-A configuration.
2. For a given dataset used for model development, performance will be best when the same dataset is used for model evaluation. For example, performance will be better in the ForceID-A → ForceID-A configuration than the ForceID-A → AIST configuration.
3. For a given dataset used for model evaluation, performance will be best when the same dataset is used for model development. For example, performance will be better in the ForceID-A → ForceID-A configuration than the AIST → ForceID-A configuration.
4. Hypotheses 1−3 will be explained by differences in gait patterns between datasets that relate to sample selection, experimental conditions or both.

## 2. Methods

### 2.1. Overview of experimental parts

There were three experimental parts, each entailing a different method for configuring the datasets for model development and evaluation (figure 1). Part I entailed same-dataset configurations, whereby models were trained, validated and tested on separate groups of individuals from the same dataset. This served as the reference method, reflecting the extent to which distinguishing features of gait were common among groups from the same population sample walking in the same set of experimental conditions. Its practical utility is limited to applications where the model deployment setting is known *a priori* and only data from that setting are available for model development. Part II entailed ‘mixed-dataset’ configurations, whereby models were trained on individuals from a reference dataset, as well as individuals from at least one other dataset, before being validated and tested on individuals from the reference dataset. Relative to part I, this part reflected the extent to which distinguishing features of gait among individuals from at least one external setting was beneficial or detrimental for distinguishing between individuals from a reference setting. From a practical perspective, it is the same as part I except data from multiple settings are available for model development. Part III entailed ‘cross-dataset’ configurations, whereby models were trained and validated on individuals from at least one dataset and then tested on individuals from a novel dataset. This part reflected the extent to which distinguishing features of gait were common among individuals from different population samples walking under different sets of experimental conditions. It is practically relevant in most applications because it does not assume knowledge of the deployment setting and shows the ability of models to generalize to a novel setting.

### 2.2. Datasets: description

Each dataset contains repeated measures of bilateral three-dimensional ground reaction forces and two-dimensional centre of pressure coordinates. These measures were acquired from force platforms embedded in series at the centre of the level and approximately 10 m long walkways inside gait analysis laboratories. The datasets differed in terms of sample selection (e.g. population demographics, number of individuals and number of samples per individual) and experimental conditions because they were acquired in independent laboratories for different purposes. Table 1 reports the key details of the experimental design for each dataset.

**Table 1.** Key details of experimental design for each dataset used in this study. S = slower than preferred speed, P = preferred speed, F = faster than preferred speed, and NA = not applicable.

Dataset	Location	Time between sessions	Footwear	Speeds
AIST [25]	Japan	NA	Barefoot	P
Gutenberg [26]	Germany	≤ 16 months	Barefoot	P
GaitRec [27]	Austria	< 1 day	Personal	S, P, F
ForceID-A [24]	Australia	3−14 days	Personal	S, P, F

AIST contains data from healthy individuals acquired over six years of research at the National Institute of Advanced Industrial Science and Technology in Japan. Individuals completed a single measurement session of barefoot walking at self-selected preferred speed. Gutenberg contains data from healthy individuals acquired over seven years of gait analysis research at Johannes Gutenberg-University Mainz in Germany. It is an accumulation of 10 data subsets that were used in separate studies. Across all subsets, 253 individuals completed a single measurement session, 42 individuals completed six sessions within a single day, eight individuals completed eight sessions within two weeks and 47 individuals completed two sessions within 7−16 months. All individuals walked barefoot at self-selected preferred speed. GaitRec contains data from both healthy and injured individuals acquired over several years of clinical practice at an Austrian rehabilitation centre. The healthy individuals were included in this study because the purpose was to investigate the variation in normal gait in the first instance. Each individual completed three−six sessions on the same day, with each session entailing walking at three self-selected speeds (preferred, slower than preferred and faster than preferred) in two shod conditions (barefoot and personal footwear). Only shod walks were included in this study, so in total there were two shod datasets and two barefoot datasets. ForceID-A contains data from healthy individuals acquired at the University of Adelaide in Australia. The private version was utilized because it contains nine more individuals compared with the public version (those who did not consent to their data being published). It was principally designed for research on the utility of gait recognition models in security applications and thus has the fewest experimental constraints. Participants completed two sessions of walking (3−14 days apart) at three self-selected speeds (preferred, slower than preferred and faster than preferred) in personal footwear. It was noted that approximately 40% of individuals wore different footwear in session two compared with session one. In most of these cases, the footwear worn in session two was of a different type.

### 2.3. Datasets: preparation

**Figure 2.**
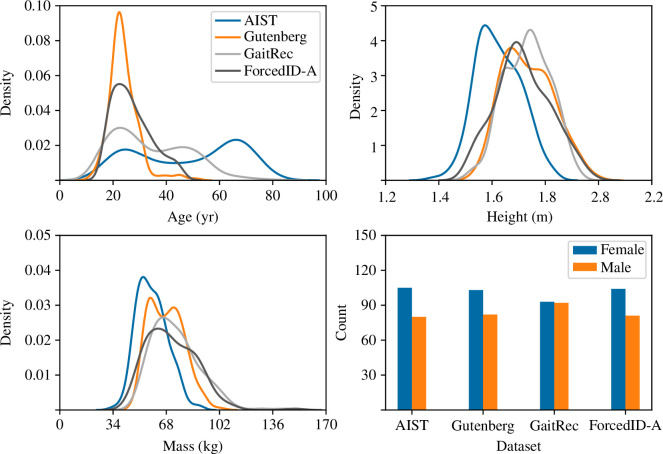
Distributions of age, height, mass and sex in each of the datasets after the number of individuals and number of samples per individual were balanced. Age, height and mass distributions were estimated via kernel density estimation.

A gait sample was defined as a sequence of two successive footsteps (i.e. stance phases) measured from a single pass along a walkway. The public version of GaitRec was used, whereas the other datasets were refined using semi-automated methods to detect samples where at least one force platform had (i) partial foot contact or multiple foot contacts; (ii) signal artefact; or (iii) signal offset due to drift. For AIST and Gutenberg, samples that met criteria (i) or (ii) were removed and samples that met criteria (iii) were calibrated. For ForceID-A, samples that met any of the criteria were removed. Details on these processes can be found in electronic supplementary material, S1.1. Afterwards, depending on the dataset, there were 193−350 individuals, 2734−8819 samples and 1−122 samples per individual (table 2). Given that gait recognition has been shown to be more difficult on force platform datasets with more individuals and fewer samples per individual, the datasets were ‘balanced’ to ensure that these characteristics did not affect model learning and performance in response to different datasets [3]. This was done by extracting 10 random samples from each of the 185 individuals in each dataset with at least 10 samples. The selected number of individuals and samples per individual was considered a trade-off. Retaining individuals was prioritized to avoid overly optimistic appraisals of model performance. Figure 2 shows the distributions of age, height, body mass and sex in the balanced datasets.

**Table 2.** The number of individuals and number of samples per individual in each dataset. A sample was defined as a sequence of two successive stance phases measured from a single pass along a walkway.

Dataset	No. of individuals	No. of samples	No. of samples per individual
AIST [25]	297	2734	1−10
Gutenberg [26]	350	8819	7−122
GaitRec (healthy shod) [27]	208	6047	9−47
ForceID-A [24]	193	5587	19−30
Total	1048	23 187	1−122
Total after balancing	740	7400	10

The method for preparing inputs for gait recognition models from raw ground reaction force and centre of pressure signals (i.e. ‘data pre-processing’) was as follows. For a given force platform:

1. Across all signals, the directions of medio-lateral and antero-posterior components were standardized. Measurement units were standardized for the ground reaction force (Newton, N) and centre of pressure (metre, m).
2. All signals were downsampled to 250 Hz, as this was the minimum sampling rate across all datasets.
3. The stance phase was initially defined as the period within which the raw vertical ground reaction force was above 50 N.
4. For the ground reaction force, the stance phase was retained with a few additional frames at each end to avoid edge effects with interpolation. For the centre of pressure, 90% of the stance phase was retained. The removal of 5% of the total number of frames at each end avoided inaccuracies in the centre of pressure at low force values [28].
5. All signals were low-pass filtered to minimize high-frequency noise (fourth-order bi-directional Butterworth, cut-off frequency 30 Hz). This is standard practice when processing ground reaction force and centre of pressure signals obtained from force platforms during walking gait [29].
6. Using linear interpolation, the times at the start and end of the stance phase where the filtered vertical ground reaction force equalled 50 N were defined. Filtered ground reaction force and centre of pressure signals were linearly interpolated to 100 evenly spaced points between these times. Normalizing the signals to a fixed length of 100 frames reduced computational load and enabled simpler model design while ensuring sufficient information content [30].
7. After training, validation and test sets were defined (§2.7), each set was standardized via *z*-score normalization. For a given frame, the measure was standardized using the mean and standard deviation across all measures in the training set at that frame.

### 2.4. Problem formulation

The gait recognition task requires detecting features of gait samples that show individual differences yet remain consistent over time. This was done via metric learning: the learning of parameters 𝜽 for a machine learning model $f_{\theta }$ that defines a set of features (i.e. a feature space) where samples from the same individual are closer (i.e. more similar) than samples from different individuals according to a distance metric. For a given query gait sample in a training set $x_{q}$ and a set of $N_{train}$ prior samples $x_{i}$ with corresponding identity labels $l_{i}$, $\{(x_{i},l_{i}){\}}_{i=1}^{N_{train}}$, the feature space was defined such that, for all i,


(1) $$\begin{array}{r} \left\|f_{\theta }(x_{q})-f_{\theta }(x_{r})\right\|<\left\|f_{\theta }(x_{q})-f_{\theta }(x_{i})\right\|\;,l_{q}=l_{r}\;,l_{i}\neq l_{r}\;. \end{array}$$

The parameters 𝜽 were updated via sequential optimization by iterating over subsets partitioned from the training set referred to as batches 𝐗. Specifically, the loss (i.e. cost) of the model was calculated using the batch hard triplet margin loss function [31]. The loss was minimized using the AMSGRAD optimization function [32]. Three different batch sizes, $N_{X}=\{128,256,512\}$, were implemented and the results for $N_{X}=512$ were presented as it led to the best performance (electronic supplementary material, table S5).

### 2.5. Performance evaluation

**Figure 3.**
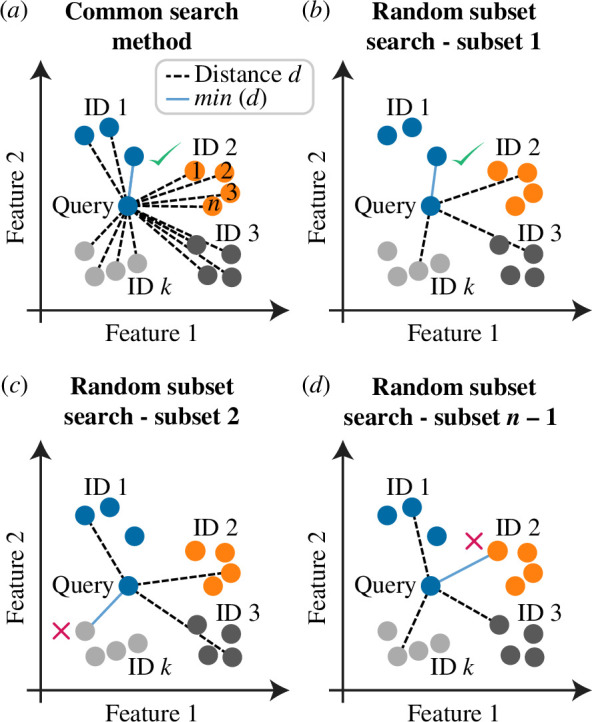
Illustration of how predictions are generated, and thus performance is measured, using the common method for searching the feature space compared with random subset search. (*a*) With k individuals (IDs) and n samples per individual, the common method entails a single search for the query sample based on all $N_{eval}$ prior gait samples ($k=4,n=4,N_{eval}=15$). The ID label associated with the prior sample that is the minimum distance from (i.e. most similar to) the query sample is predicted. In this case, accuracy =100%. (*b–d*) In random subset search, n−1=3 searches are conducted, each based on a subset containing one random prior sample per individual. In this case, accuracy =33%.

Gait recognition performance was evaluated in terms of the ability of the models to retrieve correct identity labels for novel individuals. First, features were extracted from gait samples in a given validation or test set using $f_{𝜽}$. For a given query gait sample, a prediction was generated by comparing its features with those of prior gait samples (i.e. searching the feature space) according to the distance metric and retrieving the label of the closest prior sample. The prediction was correct if the retrieved label matched the ground-truth label of the query sample. The performance metric was rank-1 accuracy (referred to herein as accuracy): the percentage of correct predictions over total number of predictions. To search the feature space to generate predictions, this study implemented a difficult method proposed in a previous study, referred to herein as ‘random subset search’ [24] (figure 3). In short, for a given query sample in a balanced validation or test set with k individuals and n samples per individual, there are n−1 prior samples from the query individual and n prior samples from other individuals. Typically, a single search is performed across all $N_{eval}$ prior samples to generate a single prediction. In random subset search, n−1 searches are performed to generate n−1 predictions. Each search is based on a subset comprising one random prior sample per individual. This simulates a challenging initial deployment scenario wherein a set of individuals pass through an area once and then one of them (the query individual) passes through a second time to be recognized.

### 2.6. Neural network architectures

The types of machine learning models used in this study were artificial neural networks. Artificial neural networks are well suited to metric learning and have been the most widely successful for gait recognition [3,9,24]. To determine if the effects of dataset configurations on model performance were moderated by model design, 20 different architectures of varying types and depths (up to six hidden layers) were implemented. The architecture types were as follows:

— fully connected,
— convolutional,
— convolutional uni-directional long short-term memory,
— convolutional bi-directional long short-term memory,
— convolutional transformer, and
— transformer.

Previous studies have found success at force platform-based gait recognition using simple architectures [22,24]. Hence, the design principle in this study was to start simple (e.g. one-layer fully connected neural network) and then incrementally add complexity using standardized modular components as building blocks. Fivefold cross-validation was conducted to obtain robust appraisals of model performance and the results report mean accuracy on test data. Details on model design, hyper-parameter settings and implementation can be found in electronic supplementary material, S1.2 and S1.3, as well as tables S1–S4.

### 2.7. Dataset configurations

This section describes the three methods used to configure individuals from each dataset into training, validation and test sets for fivefold cross-validation. Within the constraints of these methods, all possible configurations of the datasets were implemented.

**Figure 4.**
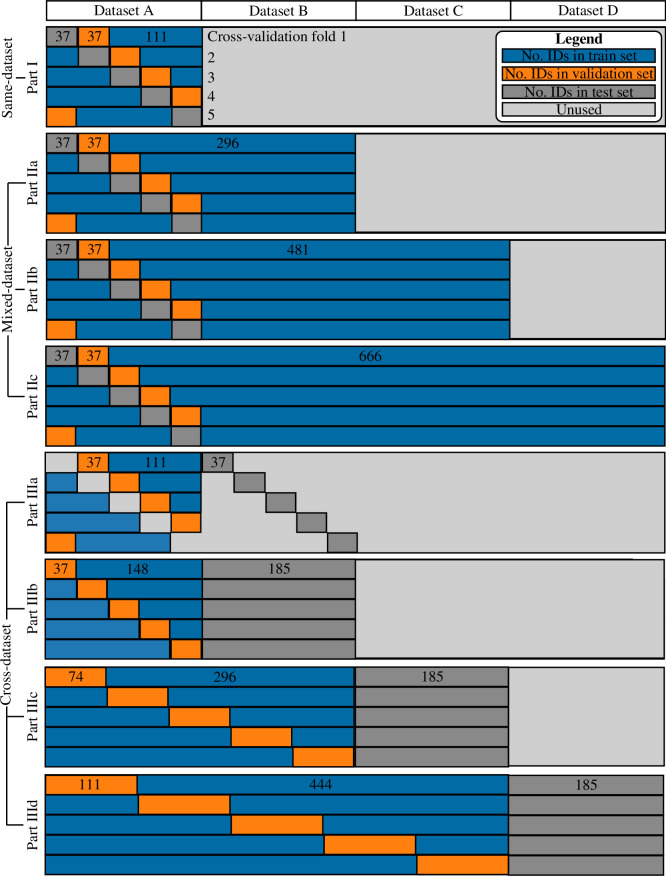
Illustration of the three methods used to configure individuals into training, validation and test sets over fivefold cross-validation. Each dataset is denoted as a variable (e.g. dataset A) rather than named specifically (e.g. AIST) because all possible configurations of the datasets were implemented within the constraints of these methods. For example, in part I, dataset A could be AIST, Gutenberg, GaitRec or ForceID-A.

For the same-dataset configurations in part I, of the 185 individuals in a given dataset, 111 were included in the training set, 37 were included in the validation set and 37 were included in the test set (figure 4; part I). For the mixed-dataset configurations in part II, three sub-parts were implemented to incorporate two, three and four datasets (figure 4; part II):

- *Two datasets.* Models were trained on 296 individuals comprising the training set from part I plus 185 individuals from another dataset. They were then validated and tested using the validation and test sets from part I (this facilitated a controlled comparison of the effect of altering the training set).
- *Three datasets.* Models were trained on a total of 481 individuals comprising the training set from part I plus 370 individuals from two other datasets. They were then validated and tested using the validation and test sets from part I.
- *Four datasets.* Models were trained on a total of 666 individuals comprising the training set from part I plus 555 individuals from three other datasets. They were then validated and tested using the validation and test sets from part I.

For the cross-dataset configurations in part III, four sub-parts were implemented to incorporate two, three and four datasets (figure 4; part III):

- *Two datasets.* Using the cross-validation folds defined in part I, models were trained and validated on one dataset and then tested on another dataset. This configuration facilitated direct comparisons with part I.
- *Two datasets.* Of the 185 individuals in one dataset, 148 were included in the training set and 37 were included in the validation set. All 185 individuals from another dataset were included in the test set.
- *Three datasets.* Of the 370 individuals in two datasets, 296 were included in the training set and 74 were included in the validation set. The individuals were shuffled prior to being allocated to training and validation sets to ensure that each set contained individuals from each dataset. All 185 individuals from a third dataset were included in the test set.
- *Four datasets.* Of the 555 individuals across three datasets, 444 were included in the training set and 111 were included in the validation set. The individuals were shuffled prior to being allocated to training and validation sets to ensure that each set contained individuals from each dataset. All 185 individuals from the remaining dataset were included in the test set.

### 2.8. Explainable artificial intelligence

In this study, two XAI methods of different types were implemented to examine if certain demographic attributes and experimental conditions helped to define variation in gait. Uniform manifold approximation and projection (UMAP) is a dimension reduction technique that can be used to visualize high-dimensional data in two dimensions [33]. This technique was applied to features extracted from gait samples to see if gait recognition models learnt to characterize gait samples according to demographic attributes and experimental conditions. Based on comparisons in performance between architectures, one architecture was selected to be re-implemented over fivefold cross-validation using training, validation and test sets so that each contained a uniform distribution of individuals from all four datasets (number of individuals = {444,148,148}, respectively). In each cross-validation fold, samples from the training set were processed by the trained model and their features were input into the UMAP function [33]. The desired separation between close points in the feature space (‘min-dist’ parameter) was zero to enable unrestrained clustering. The number of neighbours considered when approximating the local metric (‘n_neighbors’ parameter) was 100, based on the qualitative inspection of plots with different settings (electronic supplementary material, S7).

Occlusion sensitivity analysis is a method for estimating the importance that a machine learning model places on certain input features (e.g. pre-processed force platform measures) when making predictions [34]. Based on comparisons in performance between architectures, one architecture was selected to be re-evaluated on test sets from part I with sections (i.e. windows) of consecutive input feature values replaced with zero. This indicated if models developed on different datasets relied on different information for decision-making. Three different window sizes, w={10,20,40}, were implemented and w=20 appeared to provide the best trade-off between eliciting substantial changes in accuracy and maintaining sufficient resolution (electronic supplementary material, S8–S10). Starting from the first 20 input features, all discrete windows were implemented across all directional components of the ground reaction force and centre of pressure.

## 3. Results

The neural network architectures were ranked across all 72 dataset configurations and the results for the top five architectures are summarized herein. The method for ranking the architectures and remaining results (including accuracy tables) are in electronic supplementary material, S2.

### 3.1. Part I versus part II

**Figure 5.**
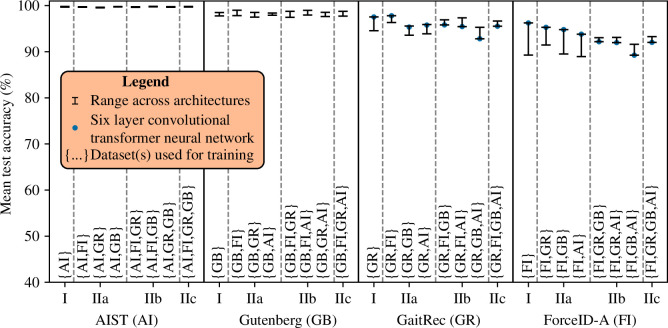
Performance of gait recognition models in part I (same-dataset configurations) compared with part II (mixed-dataset configurations). The *x*-axis labels specify the dataset used for testing and the *x*-axis ticks specify the experimental part. Within each part, a specific experiment is referenced by the notation ‘dataset used for testing–experimental part–{Dataset(s) used for training}’. For example, ‘AIST–IIa–{AI,FI}’ refers to the accuracy obtained on AIST in part IIa when individuals from AIST and ForceID-A were used for training.

Gait recognition models were highly effective in part I when trained, validated and tested on the same dataset (figure 5). Specifically, accuracy was 100% on AIST, 98 to 99% on Gutenberg, 95 to 98% on GaitRec and 89 to 96% on ForceID-A (ranges indicate variation due to model architecture). In part II, for a given dataset used for testing, adding other datasets for training had little effect on accuracy, with minor increases or decreases depending on the specific dataset configuration and architecture (figure 5). For example, on GaitRec and ForceID-A, the accuracy of the six-layer convolutional transformer neural network decreased (-2.0% and -4.2%, respectively), whereas the accuracy of other architectures increased overall (+0.24% to +3.2% depending on the architecture).

### 3.2. Part I versus part IIIa–b

**Figure 6.**
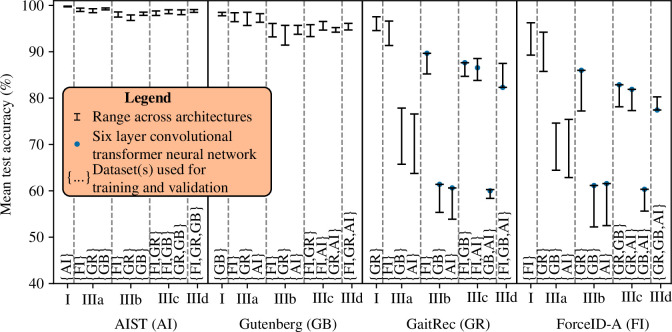
Performance of gait recognition models in part I (same-dataset configurations) compared with part III (cross-dataset configurations). The *x*-axis labels specify the dataset used for testing and the *x*-axis ticks specify the experimental part. Within each part, a specific experiment is referenced by the notation ‘dataset used for testing–experimental part–{dataset(s) used for training and validation}’. For example, ‘AIST–IIIa–{FI}’ refers to the accuracy obtained on AIST in part IIIa when individuals from ForceID-A were used for training and validation.

For a given dataset used for training and validation in part I, using a different dataset for testing in part IIIa often resulted in higher accuracy. The most prominent example was that models trained, validated and tested on ForceID-A in part I achieved lower accuracy than models trained and validated on ForceID-A and tested on AIST, Gutenberg or GaitRec in part IIIa (−3.1 to -10%, −2.2 to -8.8% and −0.38 to -2.7%, respectively; figure 6).

For a given dataset used for testing in part I, models almost always achieved lower accuracy when a different dataset was used for training and validation in parts IIIa and IIIb. From part I to part IIIa and then part IIIa to part IIIb, decreases in accuracy were small-to-moderate on AIST (≤1.3% then ≤2.5%) and Gutenberg (≤2.1% then ≤4.5%), yet often much larger on GaitRec (≤31% then ≤16%) and ForceID-A (≤27% then ≤14%) (figure 6). The large decreases in accuracy on GaitRec and ForceID-A were in cases where individuals from AIST or Gutenberg were used for training and validation.

### 3.3. Part IIIb–d

For a given dataset used for testing in part IIIb, adding individuals from a second dataset (part IIIc) and a third dataset (part IIId) for training and validation had mixed effects on performance depending on the specific dataset configuration and architecture (figure 6). On AIST and Gutenberg, accuracy either increased or decreased slightly depending on the architecture, with slight increases on average across architectures (AIST: +0.72% then +0.32%; Gutenberg: +0.75% then +0.35%). On GaitRec and ForceID-A, when the other dataset in this pair was added, the accuracy of all architectures increased markedly (+26% on average). When AIST or Gutenberg were added, the accuracy of the six-layer convolutional transformer neural network decreased (GaitRec: -1.8% then -4.8%; ForceID-A: -2.3% then -4.9%), whereas the accuracy of other architectures generally increased (GaitRec: +1.3% then -0.11%; ForceID-A: +1.6% then +0.09%).

### 3.4. Explainable artificial intelligence

**Figure 7.**
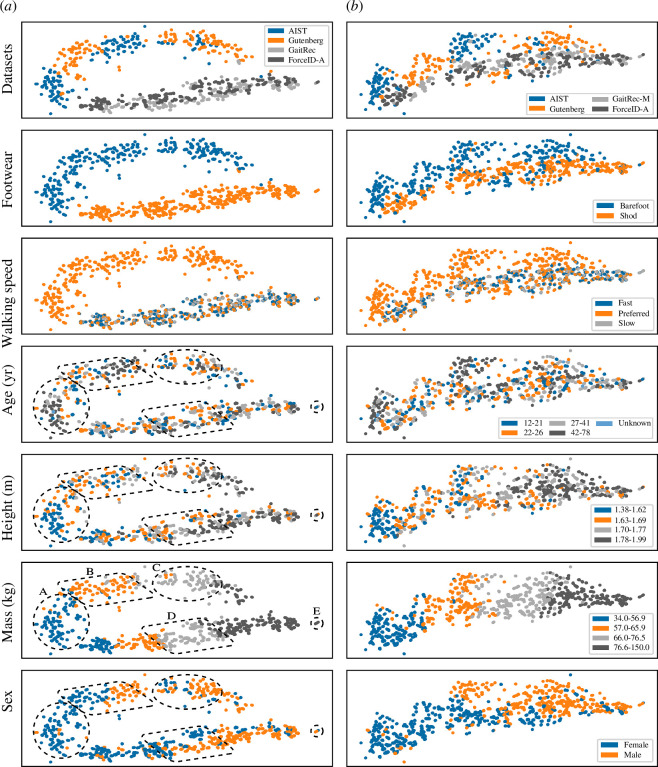
Uniform manifold approximation and projection (UMAP) plots of features extracted from gait samples by the two-layer fully connected neural network (training set—cross-validation fold two). Subplots in (*a*) were derived from a training set containing samples from each of the four datasets. Subplots in (*b*) were derived from a training set containing a different version of GaitRec (GaitRec-M) that also included barefoot samples. Samples are colour coded according to datasets, experimental conditions and quartiles in demographic attributes. Based on visual inspection, certain groups with body mass in the same quartile (roughly indicated as regions A–D) or even narrow range (region E) appeared to be mapped separately according to sex, height or both.

Despite the two-layer fully connected neural network being ranked second overall by a narrow margin, it performed most consistently across all dataset configurations and had a simple design. Hence, it was used to apply the XAI methods. Two-layer fully connected neural network models trained, validated and tested on individuals from all four datasets for the UMAP analysis achieved 95% accuracy. They also appeared to learn similar sets of gait features from the training set in each cross-validation fold (electronic supplementary material, S7). For example, the subplots in figure 7*a* show a UMAP of features extracted from gait samples in the training set from cross-validation fold two. The dataset, footwear and walking speed subplots show that there was a significant vertical shift between samples from individuals who walked barefoot at self-selected preferred speed (AIST and Gutenberg) compared with individuals who walked in personal footwear at three self-selected speeds (GaitRec and ForceID-A). A post hoc experiment was conducted to determine if the vertical shift could be attributed to footwear conditions. The same architecture was re-implemented with a different version of GaitRec, named GaitRec-M, that contained shod and barefoot gait samples as opposed to shod only. The subplots in figure 7*b* show that using GaitRec-M reduced the vertical shift.

Figure 7 also shows that gait samples were distributed according to differences in demographic attributes. The mass, sex and height subplots indicate correlations between these attributes and the mapping of gait samples along the horizontal dimension (with samples from heavier individuals, males and taller individuals towards the right). Samples from AIST and GaitRec were furthest apart in the horizontal dimension in figure 7 and had the greatest pair-wise difference in body mass, sex and height distributions in figure 2. The mass, sex and height subplots in figure 7 were visually inspected in further detail to determine if there were effects of sex and height independent of body mass. It was found that certain groups with body mass in the same quartile (roughly indicated as regions A–D) or even narrow range (region E) were mapped separately according to sex, height or both. The only noteworthy observation for age was that there were many individuals from AIST aged 42−78 years towards the bottom of region A and the right of region B.

**Figure 8.**
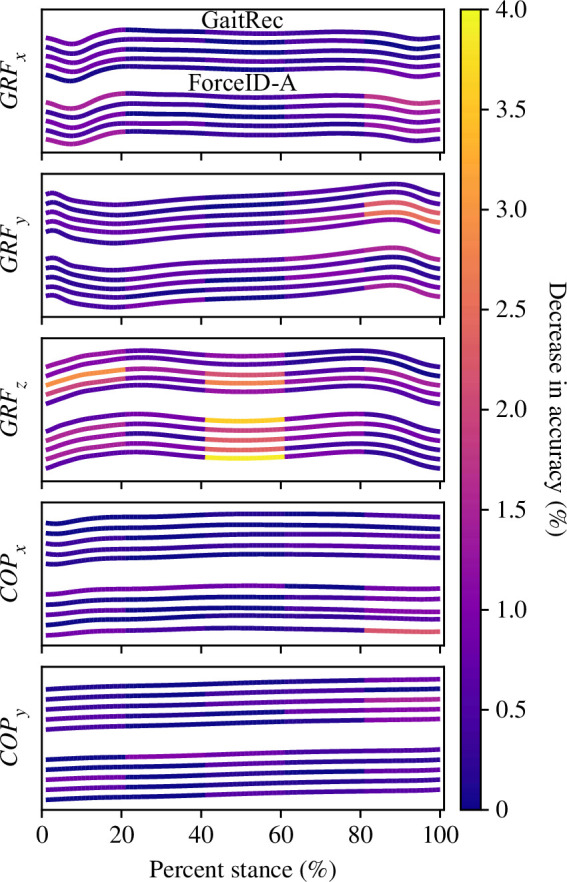
Mean ground reaction force (GRF) and centre of pressure (COP) measures across left footsteps (i.e. stance phases) in test sets from part I (directional components: medio-lateral x, antero-posterior y and vertical z). For each directional component and dataset, a line represents a cross-validation fold. Measures are colour coded at discrete windows spanning 20% of the stance phase. The colour map reflects the decrease in accuracy of the two-layer fully connected neural network model in response to the input feature values at these windows being replaced with zero.

Results from the occlusion sensitivity analysis differed depending on the dataset used to develop and evaluate two-layer fully connected neural network models. Models trained, validated and tested on AIST or Gutenberg were insensitive to the occlusion of input features (electronic supplementary material, S9). Conversely, models trained, validated and tested on GaitRec or ForceID-A were mildly sensitive to the occlusion of certain ground reaction force components in certain sub-phases of stance (figure 8). The most sensitive periods were the vertical ground reaction force during mid-stance (40 to 60%) and initial stance (0 to 20%), with changes in accuracy of −0.54 to -3.9% and −0.60 to -2.9% (respectively) depending on the cross-validation fold. These were followed by the antero-posterior ground reaction force during terminal stance (80 to 100%), with changes in accuracy of −0.15 to -2.5% depending on the cross-validation fold.

## 4. Discussion

Applying AI methods such as machine learning to force platform data for gait recognition is a promising way to personalize gait analysis in healthcare and security. Yet, previous studies using this approach could be biased towards a specific demographic or set of experimental conditions because models were developed and evaluated on either the same individuals or the same dataset. This study aimed to determine if model performance is affected by using data from different sources (i.e. datasets) and if certain demographic attributes and experimental conditions help to define gait variation. The overarching finding was that gait recognition performance differed markedly depending on the dataset(s) selected for model development and evaluation. This indicates that the extent of individual variation in gait, and the ability of gait recognition models to detect such variation, depend on the nature of the data. Perhaps this is why there are conflicting claims regarding the individuality of gait, and thus the applicability of gait recognition models. For example, state-of-the-art vision-based models achieve much higher performance when developed and evaluated on small and confined indoor datasets compared with large real-world datasets [9,35]. The wide range of performance in this study implies that general claims regarding the individuality of gait should not be made based on a single dataset (or configuration of datasets).

The results provided mixed evidence for hypotheses 1−3 as there were several nuanced performance trends. Hypothesis 3 was supported in that, for a given dataset used for model evaluation, performance was almost always best when the same dataset was used for model development. The most significant finding in support of hypothesis 3 was that models developed and evaluated on (discrete) individuals from GaitRec or ForceID-A performed markedly better than models developed on individuals from AIST or Gutenberg and evaluated on individuals from GaitRec or ForceID-A (‘trend A’). In other words, models that learnt to distinguish between individuals from Japan or Germany who walked barefoot at self-selected preferred speed were relatively ineffective at distinguishing between individuals from Austria or Australia who walked in personal footwear at three self-selected speeds. Mixed evidence was provided for hypothesis 2 in that, for a given dataset used for model development, performance was sometimes better when another dataset was used for model evaluation. For example, models developed and evaluated on (discrete) individuals from GaitRec or ForceID-A achieved high performance, yet models developed on individuals from GaitRec or ForceID-A and evaluated on individuals from AIST or Gutenberg achieved even higher performance (‘trend B’). In other words, models that learnt to distinguish between individuals from Austria or Australia who walked in personal footwear at three self-selected speeds were *more* effective at distinguishing between individuals from Japan or Germany who walked barefoot at self-selected preferred speed. Finally, mixed evidence was provided for hypothesis 1 in that adding individuals from external datasets for model development often conferred negligible benefit or slightly decreased performance. One key finding was that adding individuals from GaitRec for model development markedly improved performance when evaluating individuals from ForceID-A, and vice versa (‘trend C’).

Mixed evidence was provided for hypothesis 4 as the main trends indicated that differences in gait were sometimes greater within datasets (trend B) than between datasets (trends A and C). Based on comparisons in sample selection and experimental conditions, these trends could not be explained by differences in population and did not appear related to differences in demographic attributes. Rather, they appeared related to footwear and walking speed conditions. Footwear and walking speed were the only two reported factors that differed notably in GaitRec and ForceID-A compared with AIST and Gutenberg. Also, altering the distribution of footwear in GaitRec reduced the degree of distribution shift between gait features extracted from each pair of datasets using two-layer fully connected neural network models. The greater variation in footwear and walking speed in GaitRec and ForceID-A probably resulted in greater ratios of intra- to inter-individual variation in gait in these datasets [36,37]. In turn, models developed on these datasets focused on specific ground reaction force components and stance sub-phases (based on the occlusion sensitivity analysis) to encode subtle features of gait that were not only independent of the population but also largely independent of footwear and walking speed [38,39]. Conversely, models developed on AIST and Gutenberg focused on broader aspects of the ground reaction force and centre of pressure, encoding features of gait that were mostly specific to barefoot walking at a narrow walking speed range. This suggests that force platform-based gait recognition models must be exposed to variations in footwear and walking speed during development to effectively characterize novel individuals across footwear types (including barefoot) and walking speeds.

Subtler performance trends pointed towards additional sources of variation in gait within and between datasets. The systematically lower performance of models evaluated on ForceID-A compared with GaitRec, and Gutenberg compared with AIST, could be related to differences in the number and timing of measurement sessions. For example, individuals in ForceID-A completed two sessions separated by 3−14 days, whereas individuals in GaitRec completed three−six sessions on the same day. As such, only ForceID-A had intra-individual variation in footwear, probably resulting in a greater ratio of intra- to inter-individual variation in gait. Another subtle trend was the incompatibility of AIST and GaitRec, as evidenced by the lower performance of models evaluated on individuals from AIST when individuals from GaitRec were used for model development (as opposed to individuals from another dataset), and vice versa. Given that two-layer fully connected neural network models developed on all four datasets learnt gait features related to body mass, sex and height (based on the UMAP analysis), shifts in the distributions of these attributes may have caused shifts in the distributions of gait features learnt by various types of models in the main experiments. Interestingly, gait samples did not appear to be characterized by age in this study, despite prior works showing that certain manually defined gait features differ between groups according to age [40]. Also, shifts in distributions of age between datasets did not markedly affect their compatibility for gait recognition. It is possible that there were distinguishing features of gait that depended on age but were not detected by the two-layer fully connected neural network models or were not observable in the UMAP plots. It is also possible that previously identified effects of age are confounded by combined effects of body mass, sex and height or do not generalize to entire gait patterns. Overall, the findings from this study suggest that footwear, walking speed, body mass, sex, height and possibly other time-dependent factors interact to define variation in gait at multiple levels.

Previous studies in healthcare and security have shown variations in gait according to the factors identified in this work. However, studies oriented towards healthcare predominantly modelled group-level variation in gait according to one primary factor while controlling or adjusting for covariate and confounding factors [7,15,36,37]. For example, numerous studies compared spatio-temporal gait features between groups according to sex, accounting for possible interactions of walking speed and footwear through experimental controls or statistical adjustments [15]. Studies following this approach often leveraged high-quality gait measures from specialized measurement instruments. However, they required *a priori* assumptions regarding potential interactions between factors, leading to the use of small and confined datasets and simple (univariate) statistical models. Studies oriented towards security have predominantly altered the representation of a specific factor to a gait recognition model (through dataset, problem formulation, pre-processing method or model design) and then measured the effect on its performance [24,41–44]. This approach has typically relied on low-quality gait measures from commercial off-the-shelf vision sensors as a trade-off to enable the use of large and diverse datasets and complex (multivariate) models. Hence, previous findings regarding the effects of the factors identified in this work on individual variation in gait could be conflated with effects from factors related to body appearance. The present study built on the strengths of both approaches by implementing multivariate (machine learning) models on a large and diverse database of high-quality gait measures. Specifically, with the help of XAI, comparisons in force platform-based gait recognition model behaviour in response to data from different sources provided preliminary insight into how multiple factors interact to define individual, group and dataset-level variation in gait.

Findings from this study have two primary implications for gait analysis system engineering. First, force platforms could be deployed as stand-alone instruments with relatively few constraints on sample selection and walking conditions to enable the acquisition of large datasets in laboratories, clinics and possibly more diverse environments. This could in turn facilitate more advanced data-driven statistical analyses, initiating a positive feedback loop. The gait recognition models in this study performed very highly on individuals whose gaits were measured in experimental settings that could reasonably be expected in many clinical applications. Thus, the proposed method could be valuable for clinical gait analysis, given that vast efforts are devoted to understanding interdependent relationships between gait features (e.g. walking speed), demographic attributes (e.g. sex and body mass distribution) and pathology [7,15–17]. The method could also be applied to datasets acquired in shared or public indoor spaces to further investigate the potential of force platform-based gait recognition for security applications such as access control. Another implication of the findings is that distribution (i.e. domain) shift between training and test sets should be determined empirically rather than assumed *a priori* based on data source when engineering machine learning gait recognition models. For example, cross-dataset configurations should not be assumed to be domain generalization tasks [45]. It was only through comparisons in performance between same-dataset and multi-dataset configurations that group and dataset-level gait variation (i.e. distribution shift) could be quantified in this study. In summary, the compatibility of force platform hardware and machine learning software makes them a promising combination for practical yet personalized gait analysis.

There were a few limitations to this study. First, explanations of model performance were not exhaustive due to the limitations of the datasets. The factors provided as metadata (e.g. footwear and walking speed) were often characterized in little detail and there could be other unreported factors that affected gait patterns (e.g. clothing, emotional state and fatigue) [46–48]. Also, the dimension reduction process in UMAP could have resulted in information loss or rendered certain effects undetectable. Another limitation was that the datasets probably contain a subset of the sample characteristics, measurement protocols and experimental conditions to be encountered in many applications. For instance, many security applications would require fewer constraints on gait measurement. This could increase the amount of variation in the considered factors and introduce new factors that affect gait or its measurement (e.g. the presence of carried objects [48], partial or multiple foot contacts on a given force platform [49] and different walking directions [50]). Finally, the distribution of the AIST dataset was suspended at the time of writing, thus it could not be redistributed with the article. However, the main contribution of this study was the gait analysis method that is freely available through the public code repository. The repository is general and scalable, so it can be adapted or extended to work with an arbitrary number of force platform datasets. Despite its limitations, this study provided new insight into the nature of individual variation in human walking gait across populations and walking conditions. These insights further highlight the potential of gait as a personal functional marker and biometric.

## 5. Conclusion

This study proposed a novel gait analysis method that enables simultaneous characterization of individual, group and dataset-level variation in human walking gait. Namely, machine learning models were applied for gait recognition using different configurations of force platform datasets that each contained different population demographics and walking conditions. Trends in performance and insights from XAI indicated that footwear, walking speed, body mass, sex, height and possibly other time-dependent factors interact to affect gait variation at multiple levels. Overall, AI-based gait recognition systems that rely on force platform data show substantial promise for personalized gait analysis in healthcare and security.

## Data Availability

The code repository for this project can be found at [51] and [52]. Data is available from Figshare at [53–56]. Supplementary material is available online [57].

## References

[1] Whittle MW. 2014 Gait analysis: an introduction. Oxford, UK: Butterworth-Heinemann. See https://books.google.com.au/books?id=dYHiBQAAQBAJ.

[2] Kuo AD. 2007 The six determinants of gait and the inverted pendulum analogy: a dynamic walking perspective. Hum. Mov. Sci. **26**, 617–656. (10.1016/j.humov.2007.04.003) 17617481

[3] Horst F, Slijepcevic D, Simak M, Horsak B, Schöllhorn WI, Zeppelzauer M. 2023 Modeling biological individuality using machine learning: a study on human gait. Comput. Struct. Biotechnol. J. **21**, 3414–3423. (10.1016/j.csbj.2023.06.009) 37416082 PMC10319823

[4] Ghai S, Ghai I, Narciss S. 2022 Auditory stimulation improves gait and posture in cerebral palsy: a systematic review with between- and within-group meta-analysis. Children **9**, 1752. (10.3390/children9111752) 36421201 PMC9689391

[5] Verghese J, LeValley A, Hall CB, Katz MJ, Ambrose AF, Lipton RB. 2006 Epidemiology of gait disorders in community‐residing older adults. J. Am. Geriatr. Soc. **54**, 255–261. (10.1111/j.1532-5415.2005.00580.x) 16460376 PMC1403740

[6] Sanders JB, Bremmer MA, Deeg DJH, Beekman ATF. 2012 Do depressive symptoms and gait speed impairment predict each other’s incidence? A 16-year prospective study in the community. J. Am. Geriatr. Soc. **60**, 1673–1680. (10.1111/j.1532-5415.2012.04114.x) 22905679

[7] Andrews AW, Vallabhajosula S, Boise S, Bohannon RW. 2023 Normal gait speed varies by age and sex but not by geographical region: a systematic review. J. Physiother. **69**, 47–52. (10.1016/j.jphys.2022.11.005) 36528509

[8] Wren TAL, Tucker CA, Rethlefsen SA, Gorton GE III, Õunpuu S. 2020 Clinical efficacy of instrumented gait analysis: systematic review 2020 update. Gait Posture **80**, 274–279. (10.1016/j.gaitpost.2020.05.031) 32563727

[9] Sepas-Moghaddam A, Etemad A. 2023 Deep gait recognition: a survey. IEEE Trans. Pattern Anal. Mach. Intell. **45**, 264–284. (10.1109/TPAMI.2022.3151865) 35167443

[10] Kirtley C. 2006 Clinical gait analysis: theory and practice. Amsterdam, The Netherlands: Elsevier. See https://books.google.com.au/books?id=dF4z51oyTsEC.

[11] Nixon MS, Carter JN, Cunado D, Huang PS, Stevenage SV. 1999 Automatic gait recognition. In Biometrics (eds AK Jain, R Bolle, S Pankanti), pp. 231–249. Boston, MA: Springer. (10.1007/0-306-47044-6_11)

[12] Robertson DGE, Caldwell GE, Hamill J, Kamen G, Saunders NW. 2013 Research methods in biomechanics. (ed. DGE Robertson). Champaign, IL: Human Kinetics. (10.5040/9781492595809)

[13] Pinto-Fernández D, Rodriguez-Cianca D, Moreno JC, Torricelli D. 2024 Human locomotion databases: a systematic review. IEEE J. Biomed. Health Inform. **28**, 1716–1729. (10.1109/JBHI.2023.3311677) 37665701

[14] Roberts M, Mongeon D, Prince F. 2017 Biomechanical parameters for gait analysis: a systematic review of healthy human gait. Phys. Ther. Rehabil. **4**, 6. (10.7243/2055-2386-4-6)

[15] Frimenko R, Goodyear C, Bruening D. 2015 Interactions of sex and aging on spatiotemporal metrics in non-pathological gait: a descriptive meta-analysis. Physiotherapy **101**, 266–272. (10.1016/j.physio.2015.01.003) 25702092

[16] Buckley C, Alcock L, McArdle R, Rehman RZU, Del Din S, Mazzà C, Yarnall AJ, Rochester L. 2019 The role of movement analysis in diagnosing and monitoring neurodegenerative conditions: insights from gait and postural control. Brain Sci. **9**, 34. (10.3390/brainsci9020034) 30736374 PMC6406749

[17] Holanda LJ, Silva PMM, Amorim TC, Lacerda MO, Simão CR, Morya E. 2017 Robotic assisted gait as a tool for rehabilitation of individuals with spinal cord injury: a systematic review. J. Neuroeng. Rehabil. **14**, 126. (10.1186/s12984-017-0338-7) 29202845 PMC5715997

[18] Singh JP, Jain S, Arora S, Singh UP. 2018 Vision-based gait recognition: a survey. IEEE Access **6**, 70497–70527. (10.1109/ACCESS.2018.2879896)

[19] Wang L, Ning H. 2003 Silhouette analysis-based gait recognition for human identification. IEEE Trans. Pattern Anal. Mach. Intell. **25**, 1505–1518. (10.1109/TPAMI.2003.1251144)

[20] Teepe T, Gilg J, Herzog F, Hormann S, Rigoll G. 2022 Towards a deeper understanding of skeleton-based gait recognition. In 2022 IEEE/CVF Conf. on Computer Vision and Pattern Recognition Workshops (CVPRW), New Orleans, LA, pp. 1569–1577. New York, NY: IEEE. (10.1109/CVPRW56347.2022.00163)

[21] Derlatka M, Borowska M. 2023 Ensemble of heterogeneous base classifiers for human gait recognition. Sensors **23**, 508. (10.3390/s23010508) 36617105 PMC9824449

[22] Horst F, Lapuschkin S, Samek W, Müller KR, Schöllhorn WI. 2019 Explaining the unique nature of individual gait patterns with deep learning. Sci. Rep. **9**, 2391. (10.1038/s41598-019-38748-8) 30787319 PMC6382912

[23] Barredo Arrieta A *et al*. 2020 Explainable artificial intelligence (XAI): concepts, taxonomies, opportunities and challenges toward responsible AI. Inf. Fus. **58**, 82–115. (10.1016/j.inffus.2019.12.012)

[24] Duncanson KA, Thwaites S, Booth D, Hanly G, Robertson WSP, Abbasnejad E, Thewlis D. 2023 Deep metric learning for scalable gait-based person re-identification using force platform data. Sensors **23**, 3392. (10.3390/s23073392) 37050451 PMC10099366

[25] Kobayashi Y, Hida N, Nakajima K, Fujimoto M, Mochimaru M. 2019 AIST gait database. See https://unit.aist.go.jp/harc/ExPART/GDB2019_e.html.

[26] Horst F, Slijepcevic D, Simak M, Schöllhorn WI. 2021 Gutenberg gait database, a ground reaction force database of level overground walking in healthy individuals. Sci. Data **8**, 232. (10.1038/s41597-021-01014-6) 34475412 PMC8413275

[27] Horsak B, Slijepcevic D, Raberger AM, Schwab C, Worisch M, Zeppelzauer M. 2020 GaitRec, a large-scale ground reaction force dataset of healthy and impaired gait. Sci. Data **7**, 143. (10.1038/s41597-020-0481-z) 32398644 PMC7217853

[28] Chockalingam N, Giakas G, Iossifidou A. 2002 Do strain gauge force platforms need in situ correction? Gait Posture **16**, 233–237. (10.1016/s0966-6362(02)00017-6) 12443947

[29] Yu B, Gabriel D, Noble L, An KN. 1999 Estimate of the optimum cutoff frequency for the Butterworth low-pass digital filter. J. Appl. Biomech. **15**, 318–329. (10.1123/jab.15.3.318)

[30] Burdack J, Horst F, Giesselbach S, Hassan I, Daffner S, Schöllhorn WI. 2020 Systematic comparison of the influence of different data preprocessing methods on the performance of gait classifications using machine learning. Front. Bioeng. Biotechnol. **8**, 260. (10.3389/fbioe.2020.00260) 32351945 PMC7174559

[31] Hermans A, Beyer L, Leibe B. 2017 In defense of the triplet loss for person re-identification. arXiv Preprint. (10.48550/arXiv.1703.07737)

[32] Reddi SJ, Kale S, Kumar S. 2019 On the convergence of Adam and beyond. arXiv Preprint. (10.48550/arXiv.1904.09237)

[33] McInnes L, Healy J, Saul N, Großberger L. 2019 UMAP: uniform manifold approximation and projection. J. Open Source Softw. **3**, 861. (10.21105/joss.00861)

[34] Zeiler MD, Fergus R. 2014 Visualizing and understanding convolutional networks. In Computer Vision - ECCV 2014 (eds D Fleet, T Pajdla, B Schiele, T Tuytelaars). Cham, Switzerland: Springer. (10.1007/978-3-319-10590-1_53)

[35] Zhu Z, Guo X, Yang T, Huang J, Deng J, Huang G, Du D, Lu J, Zhou J. 2021 Gait recognition in the wild: a benchmark. In Proc. of the IEEE/CVF Int. Conf. on Computer Vision (ICCV), pp. 14789–14799. New York, NY: IEEE. https://www.grew-benchmark.org.

[36] Franklin S, Grey MJ, Heneghan N, Bowen L, Li FX. 2015 Barefoot vs common footwear: a systematic review of the kinematic, kinetic and muscle activity differences during walking. Gait. Posture **42**, 230–239. (10.1016/j.gaitpost.2015.05.019) 26220400

[37] Fukuchi CA, Fukuchi RK, Duarte M. 2019 Effects of walking speed on gait biomechanics in healthy participants: a systematic review and meta-analysis. Syst. Rev. **8**, 153. (10.1186/s13643-019-1063-z) 31248456 PMC6595586

[38] Gong Z, Zhong P, Hu W. 2019 Diversity in machine learning. IEEE Access **7**, 64323–64350. (10.1109/ACCESS.2019.2917620)

[39] Wen Q, Sun L, Yang F, Song X, Gao J, Wang X, Xu H. 2021 Time series data augmentation for deep learning: a survey (ed. ZH Zhou). In 30th Int. Joint Conf. on Artificial Intelligence {IJCAI-21}, Montreal, Canada, p. 4653. San Francisco, CA: Curran Associates, Inc. (10.24963/ijcai.2021/631)

[40] Boyer KA, Johnson RT, Banks JJ, Jewell C, Hafer JF. 2017 Systematic review and meta-analysis of gait mechanics in young and older adults. Exp. Gerontol. **95**, 63–70. (10.1016/j.exger.2017.05.005) 28499954

[41] Connor P, Ross A. 2018 Biometric recognition by gait: a survey of modalities and features. Comput. Vis. Image Underst. **167**, 1–27. (10.1016/j.cviu.2018.01.007)

[42] Derlatka M, Bogdan M. 2018 Recognition of a person wearing sport shoes or high heels through gait using two types of sensors. Sensors **18**, 1639. (10.3390/s18051639) 29883389 PMC5982328

[43] Yao ZM, Zhou X, Lin ED, Xu S, Sun YN. 2010 A novel biometrie recognition system based on ground reaction force measurements of continuous gait. In 3rd Int. Conf. on Human System Interactions (HSI), Rzeszow, Poland. (10.1109/HSI.2010.5514531)

[44] Derlatka M. 2020 Time removed repeated trials to test the quality of a human gait recognition system. In Int. Conf. on Computer Information Systems and Industrial Management (eds K Saeed, J Dvorský), pp. 15–24. Cham, Switzerland: Springer. (10.1007/978-3-030-47679-3_2)

[45] Zhou K, Liu Z, Qiao Y, Xiang T, Loy CC. 2023 Domain generalization: a survey. IEEE Trans. Pattern Anal. Mach. Intell. **45**, 4396–4415. (10.1109/TPAMI.2022.3195549) 35914036

[46] Park K, Rosengren KS, Horn GP, Smith DL, Hsiao-Wecksler ET. 2011 Assessing gait changes in firefighters due to fatigue and protective clothing. Saf. Sci. **49**, 719–726. (10.1016/j.ssci.2011.01.012)

[47] Adolph D, Tschacher W, Niemeyer H, Michalak J. 2021 Gait patterns and mood in everyday life: a comparison between depressed patients and non-depressed controls. Cognit. Ther. Res. **45**, 1128–1140. (10.1007/s10608-021-10215-7)

[48] Qu X, Yeo JC. 2011 Effects of load carriage and fatigue on gait characteristics. J. Biomech. **44**, 1259–1263. (10.1016/j.jbiomech.2011.02.016) 21397234

[49] Villeger D, Costes A, Watier B, Moretto P. 2014 An algorithm to decompose ground reaction forces and moments from a single force platform in walking gait. Med. Eng. Phys. **36**, 1530–1535. (10.1016/j.medengphy.2014.08.002) 25239287

[50] Courtine G, Schieppati M. 2003 Human walking along a curved path. II. Gait features and EMG patterns. Eur. J. Neurosci. **18**, 191–205. (10.1046/j.1460-9568.2003.02737.x) 12859352

[51] Duncanson KA, Horst F, Abbasnejad E, Hanly G, Robertson WSP, Thewlis D. 2024 ForceID-Study-2. See https://github.com/kayneduncanson1/ForceID-Study-2.10.1098/rsif.2024.056539657792

[52] kayneduncanson1. 2024 ForceID-Study-2: ForceID-Study-2 version 1. (10.5281/zenodo.14013383)

[53] Horst F, Slijepcevic D, Simak M, Schöllhorn WI. 2021 Gutenberg Gait Database: a ground reaction force database of level overground walking in healthy individuals (10.6084/m9.figshare.c.5311538) PMC8413275 34475412

[54] Horsak B, Slijepcevic D, Raberger AM, Schwab C, Worisch M, Zeppelzauer M. 2020 . GaitRec. A large-scale ground reaction force dataset of healthy and impaired gait. Figshare. (10.6084/m9.figshare.c.4788012) PMC7217853 32398644

[55] Duncanson K, Thwaites S, Booth D, Hanly G, Robertson W, Abbasnejad E *et al*. 2021 ForceID Dataset A. The University of Adelaide. (10.25909/14482980)

[56] Kobayashi Y, Hida N, Nakajima K, Fujimoto M, Mochimaru M. 2019 AIST Gait Database 2019 (10.25909/14482980)

[57] Duncanson KA, Horst F, Abbasnejad E, Hanly G, Robertson W, Thewlis D. 2024 Supplementary material from: Modelling individual variation in human walking gait across populations and walking conditions via gait recognition. Figshare. (10.6084/m9.figshare.c.7557355)39657792

